# 841. Evaluating If Promise Meets Practice: Use of a Novel Dual-layer Biosynthetic Wound Matrix

**DOI:** 10.1093/jbcr/irag033.280

**Published:** 2026-04-06

**Authors:** Bo Hyun Kong, Eden C Norris, Julianna Richards, Laura S Johnson, Lauren B Nosanov

**Affiliations:** Emory University School of Medicine, Atlanta, GA; Seattle Children’s Hospital, Seattle, WA; Emory University School of Medicine, Atlanta, GA; Grady Memorial Hospital / Emory School of Medicine / Morehouse School of Medicine, Atlanta, GA; Emory University School of Medicine, Atlanta, GA

## Abstract

**Introduction:**

Dual-layer biosynthetic wound matrices (BSMs) are used to protect partial-thickness wounds and donor sites while supporting re-epithelialization. Evolving practice with early adoption of technology outpaces published evidence: real-world utilization patterns, process-of-care details, and the correlation between short and long-term outcomes remain under-reported. We describe our experience and insights gained in deploying a novel BSM at a large, urban Burn center and subsequent quality improvement investigations performed to hone our practice.

**Methods:**

We performed a retrospective review of all consecutive BSM applications to partial thickness wounds in adult and pediatric patients managed by the Burn service between 5/2024-11/2024. Operative management was dictated by surgeon judgment. Common practices included fixation with staples or surgical glue; dry dressings with takedown on postoperative day (POD) 1–3 and transition to open-to-air when adherent; twice-daily silver nitrate wring-outs for persistently moist wounds; targeted windowing or early removal for non-adherence, bleeding, or infection concern; removal when flaking if uncomplicated; showering permitted once fully adherent. Data captured included patient demographics and comorbidities, injury details, operative technique (wound type/site, fixation, dressings, use of autologous skin cell suspension), complications, clinical outcomes, and long-term wound healing measures.

**Results:**

Thirty-nine patients underwent 49 BSM applications. Mean age was 38.8 ± 17.9 years; burns were predominantly due to flame (51.3%) or scald (30.8%) with median total body surface area 18.8% (IQR 23.5), and inhalation injury in 12.8%. Non-burn patients included friction injury and hidradenitis suppurativa. BSMs were applied predominantly to split thickness skin graft donor sites (85.7%), most commonly on lower extremities (right 42.9%, left 57.1%). Early events included two suspected and one confirmed infection and seven bleeding events. Application of ASCS to the wound bed underneath the BSM was performed in 59.2% of applications overall, and median treated surface area was 922 cm^2^ (IQR 700; range 120–3870). Showering post-placement was limited by historical practice patterns and patient factors; improvement was seen for admitted patients with nursing education.

**Conclusions:**

In early institutional experience, BSMs were deployed largely for donor-site management, especially on the lower extremities, with staple fixation as the dominant strategy and low infectious event counts.

**Applicability of Research to Practice:**

These granular, real-world data—spanning capture methodology, bedside care pathways, and early outcomes—can serve as a template for protocol development and prospective evaluation of epithelialization, scar quality (including laser needs), and patient-reported comfort.

**Funding for the study:**

N/A.

